# Correction to: Negative effects of iodine-based contrast agent on renal function in patients with moderate reduced renal function hospitalized for COVID-19

**DOI:** 10.1186/s12882-021-02536-2

**Published:** 2021-09-30

**Authors:** Anna Kistner, Chen Tamm, Ann Mari Svensson, Mats O. Beckman, Fredrik Strand, Magnus Sköld, Sven Nyrén

**Affiliations:** 1 Medical Radiation Physics and Nuclear Medicine, Karolinska University Hospital, 171 76 Solna, Stockholm, Sweden; 2 Department of Molecular Medicine and Surgery, Karolinska Institutet, Stockholm, Sweden; 3 Department of Radiology, Karolinska University Hospital, Solna, Stockholm, Sweden; 4 Department of Medicine Solna, Karolinska Institutet, Stockholm, Sweden; 5 Department of Respiratory Medicine and Allergy, Karolinska University Hospital, Stockholm, Sweden

**Correction to: BMC Nephrology (2021) 22:297**

**https://doi.org/10.1186/s12882-021-02469-w**

Following publication of the original article [1], the authors informed us that author Ann Mari Svensson has been incorrectly affiliated.

The author affiliation has been updated above and the original article [1] has been corrected.
